# A link between adipogenesis and innate immunity: RNase-L promotes 3T3-L1 adipogenesis by destabilizing Pref-1 mRNA

**DOI:** 10.1038/cddis.2016.323

**Published:** 2016-11-10

**Authors:** Yi-Ting Wang, Hou-Hsien Chiang, Ying-Shing Huang, Chia-Lang Hsu, Po-Jen Yang, Hsueh-Fen Juan, Wei-Shiung Yang

**Affiliations:** 1 Graduate Institute of Clinical Medicine, College of Medicine, National Taiwan University, Taipei, Taiwan; 2 Division of Endocrinology & Metabolism, Department of Internal Medicine, National Taiwan University Hospital, Taipei, Taiwan; 3 Program in Metabolic Biology, Nutritional Sciences and Toxicology, University of California, Berkeley, CA 94720, USA; 4 Department of Life Science, National Taiwan University, Taipei, Taiwan; 5 Department of Surgery, National Taiwan University Hospital, Taipei, Taiwan; 6 Center for Obesity, Lifestyle and Metabolic Surgery, National Taiwan University Hospital, Taipei, Taiwan; 7 Research Center for Developmental Biology and Regenerative Medicine, National Taiwan University, Taipei, Taiwan; 8 Graduate Institute of Medical Genomics & Proteomics, College of Medicine, National Taiwan University, Taipei, Taiwan

## Abstract

Ribonuclease L (RNase-L) is an endoribonuclease well known for its roles in innate immunity. Recently it has been shown to regulate several cellular functions by modulating the levels of specific mRNAs. In this study, we investigated whether RNase-L may regulate adipocyte functions. We showed that knockdown of RNase-L reduced 3T3-L1 adipocyte differentiation and lipid accumulation. After mRNA profiling, we found that upregulation of Pref-1 mRNA, an inhibitory regulator of adipogenesis, could explain the reduced adipocyte differentiation with RNase-L downregulation. The signaling molecules downstream to Pref-1, including focal adhesion kinase, extracellular signal-regulated kinases and SRY-box 9, were activated by RNase-L suppression. The presence of Pref-1 mRNA was detected in the mRNP complexes precipitated by anti-RNase-L antibody. Moreover, the Pref-1 mRNA decay rate was raised by elevated RNase-L ribonuclease activity. Finally, in stable cell clones with RNase-L silencing, suppression of Pref-1 mRNA by specific siRNA partially recovered the adipocyte differentiation phenotype. Consistent with our findings, meta-analysis of 45 public array datasets from seven independent studies showed a significant negative relationship between RNase-L and Pref-1 mRNA levels in mouse adipose tissues. Higher RNase-L and lower Pref-1 mRNAs were found in the adipose tissues of high-fat diet mice compared to those of ND mice. In line with this, our animal data also showed that the adipose tissues of obese rats contained higher RNase-L and lower Pref-1 expression in comparison to that of lean rats. This study demonstrated that Pref-1 mRNA is a novel substrate of RNase-L. RNase-L is involved in adipocyte differentiation through destabilizing Pref-1 mRNA, thus offering a new link among RNA metabolism, innate immunity and adipogenesis in obesity progression.

Ribonuclease L (RNase-L) is an innate immune mediator in mammalian cells. It participates in antiviral and antibacterial activities induced by type I interferon through the activation of oligoadenylate synthetase (OAS)-RNase-L-retinoic acid-inducible gene 1 pathway.^1^ Taking its antiviral action as an example, once the invading viral RNAs are recognized, OAS is activated to produce 2-5A (ppp5′A[2′p5′A]n). In turn 2-5A activates RNase-L. The activated RNase-L thus recognizes and degrades the viral RNAs not only to hinder further viral replication but also to promote a series of downstream signals, which turn on the inflammatory responses, such as interferon*α*, interferon*β*, interleukin-1*β* and tumor necrosis factor-*α* production.^2, 3, 4^ In addition to its roles in the defense against microorganisms, RNase-L, through these inflammatory responses, was also reported to be involved in the progression of islet inflammation in a type 1 diabetes mellitus animal model.^5^ The association between the mutations of human RNase-L gene and the presence of hereditary and sporadic prostate cancer were also documented.^6^ These observations suggest that RNase-L may participate in the pathogenesis of many other human diseases. Indeed, in recent years, RNase-L was also demonstrated to have diverse ribonuclease activities and degrade various specific endogenous cellular RNAs.^7^ As per the results, RNase-L is capable of modulating several cellular functions, including cellular growth, cell cycle and even tumorigenesis, by regulating Hu Antigen R or tristetraprolin mRNA abundance.^8, 9, 10^ Moreover, myogenesis is also shown to be regulated by RNase-L through destabilizing MyoD and myogenin mRNA.^11, 12^

In recent decades, obesity epidemic has led to increased prevalence of metabolic syndrome, type 2 diabetes mellitus, hypertension, dyslipidemia and cardiovascular diseases.^13^ Obesity is not simply a state of excessive fat accumulation, but is also considered as a condition with chronic low-grade inflammation, coined as metainflammation.^14, 15^ Since RNase-L has a classical role in innate immunity and has adopted some new identities as regulators of other cellular functions, we are interested in whether it may modulate adipocyte functions.

## Results

### Reduced 3T3-L1 differentiation by RNase-L knockdown

**Figure 1:**
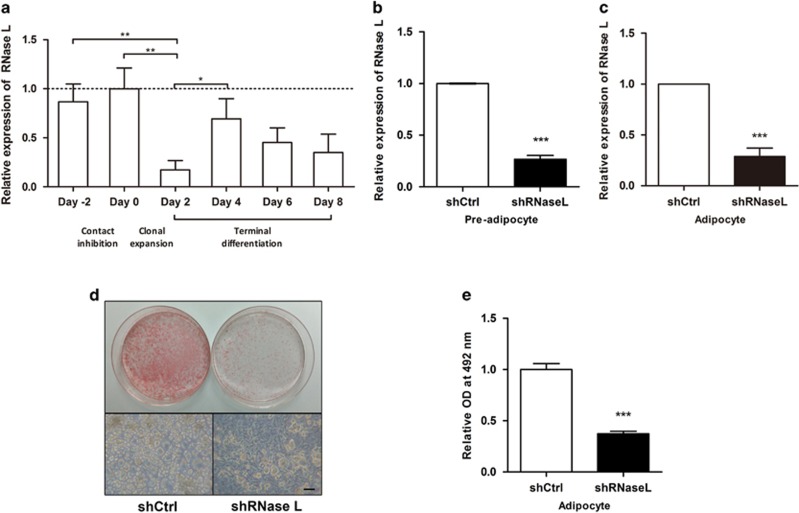
RNase-L downregulation reduced adipocyte differentiation and lipid accumulation in 3T3-L1 pre-adipocytes. (**a**) The trend of RNase-L mRNA levels during adipocyte differentiation analyzed by qPCR is shown. The RNase-L expression of Day 0 was set as 100%, and 18S rRNA was used as the reference gene. (**b**) The silencing efficiency of shRNaseL lentiviral transduction in 3T3-L1 pre-adipocytes is shown, compared to control (shCtrl). (**c**) The RNase-L gene silencing and control 3T3-L1 pre-adipocytes were subjected to differentiation, and the RNase-L differential expression of the cells is shown. (**d**) After differentiation, the lipid accumulation of RNase-L knockdown (right, shRNaseL) and control (left, shCtrl) cells were determined by Oil red O staining (upper) and bright field microscopy imaging (lower). Scale bar represents 50 μm. (**e**) The lipid content of 3T3-L1 were quantified. The data shown are mean±S.E.M. obtained in three or more independent experiments. *P<0.05, **P<0.01 and ***P<0.001

In order to investigate whether RNase-L plays a role in adipocyte differentiation, we first analyzed the changes of RNase-L expression during 3T3-L1 differentiation by qPCR. Once the differentiation is induced, the expression of RNase-L mRNA decreases (Figure 1a). At the stage of full differentiation (Day 8), the mRNA level of RNase-L was approximately 34.9% that of undifferentiated 3T3-L1 cells (Day 0). At Day 2, while the cells were still under induction medium, the expression level of RNase-L mRNA was the lowest, approximately 17.2% that of Day 0 (Figure 1a). This result suggests the expression of RNase-L was developmentally regulated in 3T3-L1 adipocyte differentiation.

We next performed gene knockdown experiments using lentivirus-mediated RNase-L shRNA (shRNaseL) in 3T3-L1 cells. In the undifferentiated 3T3-L1 cells, the RNase-L mRNA expression was down to 26.5% that of the control (Figure 1b), whereas in differentiated cells, it was down to 28.6% that of control (Figure 1c). We found that the differentiation of 3T3-L1 cells into adipocytes was clearly disturbed by RNase-L downregulation (Figure 1d). With RNase-L downregulation, the lipid content decreased to 37.4% that of the control cells (Figure 1e). These data indicated that RNase-L expression is required for adipocyte differentiation, probably in the early stage of differentiation process.

### Downregulation of RNase-L altered the expression of adipogenesis-related genes

Adipogenesis is a multistep process that involves immensely complicated and sequential transcriptional regulation of various activator and repressor genes. It can be arbitrarily divided into three stages: pre-induction phase, early phase and late phase after induction.^16^ It was plausible to propose that those up-regulated negative regulators upon RNase-L gene knockdown are the potential candidates responsible for reduced adipocyte differentiation in view of the function of RNase-L.

**Figure 2:**
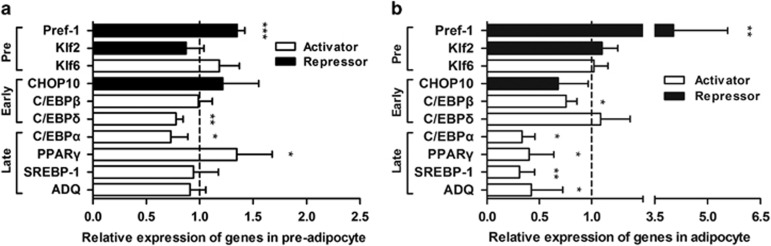
Downregulation of RNase-L altered the expression of adipogenesis-related genes. Un-differentiated and differentiated RNase-L knockdown 3T3-L1 cells were subjected to the expression analyses of adipogenesis-related genes by qPCR compared to the control cells. The differentiation process is arbitrarily divided into three stages: pre-induction (Pre), early phase (Early) and late phase (Late) after induction. (**a**) The expression patterns of various indicated genes in undifferentiated 3T3-L1 cells with reduced RNase-L gene expression compared to the controls are shown. (**b**) In differentiated 3T3-L1 adipocytes, the expression patterns of these genes with RNase-L knockdown compared to the controls are shown. White bars=activator genes; black bars=repressor genes. The bar charts shown are mean±S.E.M. obtained in three or more independent experiments. *P<0.05, **P<0.01 and ***P<0.001

Among the genes in the pre-induction phase, downregulation of RNase-L resulted in a significant and consistent increase in pre-adipocyte factor-1 (Pref-1), respectively, by 1.4-fold in un-differentiated (Figure 2a) and by 4.0-fold in differentiated 3T3-L1 cells (Figure 2b), compared to the control cells. The other regulators in the pre-induction phase were not significantly changed (Figures 2a and b). For the genes in the early phase after induction, the mRNA levels of CCAAT-enhancer-binding protein (C/EBP) *δ* and *β* were reduced, respectively, in undifferentiated and differentiated 3T3-L1 cells (Figures 2a and b) with RNase-L knockdown. For the late-phase regulatory genes, only CCAAT-enhancer-binding protein *α* was down-regulated both in undifferentiated and in differentiated 3T3-L1 cells (Figures 2a and b). Apart from this, the mRNA levels of the adipogenic master genes, including peroxisome proliferator-activated receptor *γ* (PPAR*γ*, down to 40.5%), sterol regulatory element-binding protein-1 (SREBP-1, down to 31.2%) and terminal differentiation marker adiponectin (ADQ, down to 42.4%), were significantly reduced in differentiated state (Figure 2b).

We also assayed the gene expression of the Krüppel-like factor (KLF) family members related to adipogenesis.^17^ Klf2 and Klf6, which oppositely regulate Pref-1 expression in transcription, had no significant change both in undifferentiated and in differentiated cells with reduced RNase-L gene expression (Figures 2a and b).^18, 19^ Klf9 and Klf15, which are capable of transactivating PPAR*γ* expression, were both down-regulated in differentiated RNase-L knockdown cells (data not shown).^20, 21^ Klf3, 4, 5 and 7, which serve diverse functions in adipogenesis,^17^ showed no significant alteration in RNase-L knockdown cells (data not shown).

Since Pref-1 is the most upstream repressor to adipocyte differentiation, we therefore postulated that Pref-1 upregulation could be responsible for reduced adipocyte differentiation with decrease RNase-L gene expression.

### RNase-L knockdown induced Pref-01 downstream signaling

**Figure 3:**
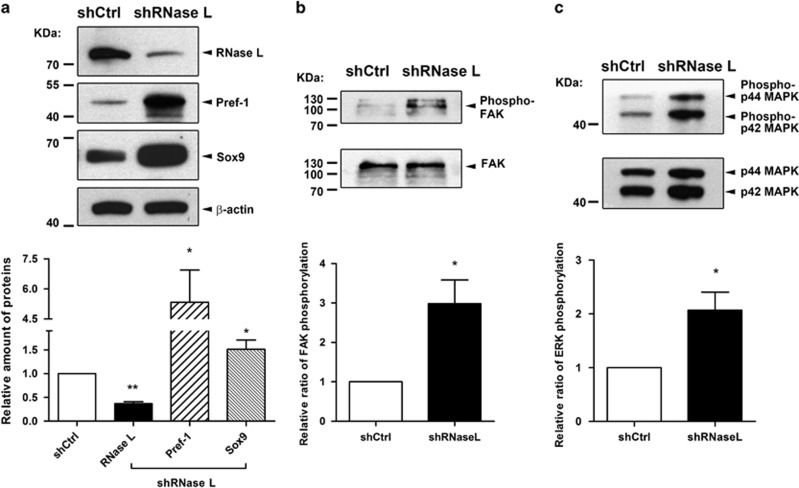
RNase-L gene silencing increased Pref-1 protein and its downstream signaling. (**a**) The protein levels of RNase-L, Pref-1 and Sox9 with RNase-L knockdown (shRNaseL) and control (shCtrl) were determined by western blotting in differentiated 3T3-L1 adipocyte and *β*-actin was used as the internal control. (**b**) The activation of FAK determined by phosphorylation of FAK is shown and total FAK was used as the control. (**c**) The activation of ERK1/2 determined by phosphorylation of ERK is shown and total ERK was used as the control. All the quantifications were based on three independent experiments. The data shown are mean±S.D. *P<0.05 and **P<0.01

To further support the potential role of Pref-1, we investigated whether Pref-1 protein was up-regulated and whether some signaling pathways downstream to Pref-1 were activated. With reduced RNase-L protein expression (by 37.2%) in differentiated 3T3-L1 adipocytes, the protein level of Pref-1 was increased by 5.3-fold compared to the control (Figure 3a). Consistent with the activation of the signaling downstream to Pref-1, the activation levels of focal adhesion kinase (FAK) and extracellular signal-regulated kinases1/2 (ERK1/2), examined by the phosphorylation ratio of the molecules, were also induced, respectively, by 3.0-fold (Figure 3b) and 2.1-fold (Figure 3c). Similarly, the downstream transcription factor SRY-box 9 (Sox9) was increased by 1.5-fold (Figure 3a), compared to the control cells. Taken together, these results demonstrated that repression of RNase-L gene expression triggered downstream signaling pathways of Pref-1, involving FAK-ERK-Sox9 activation, previously documented to be inhibitory of adipogenesis.^22, 23, 24^

### Physical association of Pref-1 mRNA with RNase-L protein

**Figure 4:**
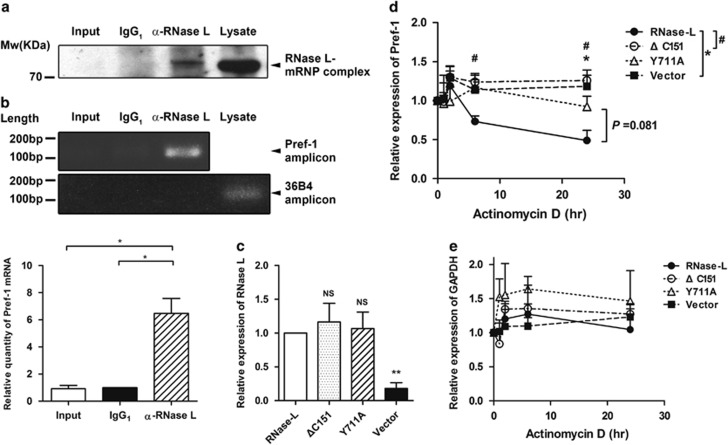
Pref-1 mRNA was recovered from RNase-L-mRNP complex and the upregulation of RNase-L enzyme activity increased Pref-1 mRNA turnover rate. (**a**) RNase-L was immunoprecipitated with mouse anti-RNase-L antibody and determined by western blotting (*α*-RNase-L). Being as the negative controls, lane 1 was the cell lysate going only through the beads (Input) and lane 2 (IgG_1_) was the lysate precipitated with non-specific IgG_1_ antibody isotype control. The whole cell lysate (Lysate) without immunoprecipitation was used as the positive control. (**b**) RT-PCR was performed to determine the presence of Pref-1 mRNA from the RNA which was extracted from each precipitates. 36B4 was used as the negative control. The PCR products were electrophoresed with 1% agarose gel and then the amplicons of Pref-1 were quantitated. (**c**) The mRNA expression levels of RNase-L in empty vector transfected (Vector), CMV-driven RNase-L-pcDNA3.1(−) (RNase-L), C-terminal 151 amino acid deletion (*Δ*C151) and Tyr711 to Ala single amino acid substitution (Y711A) transfected 3T3-L1 pre-adipocytes were compared by qPCR using RNase-L #2 (exon 4-5) primer pair. (**d**) The turnover rates of Pref-1 mRNA in these transfected 3T3-L1 pre-adipocytes are shown. (**e**) The turnover rates of GAPDH mRNA in these transfected 3T3-L1 pre-adipocytes are shown. The mRNA expression at various time points after actinomycin D treatment was examined by qPCR. All the quantifications were based on three or more independent experiments, and the mean±S.E.M. are shown. NS=not significant, *P<0.05, ^#^*P*<0.05 and **P<0.01

Since RNase-L is a ribonuclease, it is reasonable to postulate that Pref-1 mRNA could be a substrate of RNase-L and physically associated. To answer this, we immunoprecipitated RNase-L protein from 3T3-L1 pre-adipocyte cell extract, and looked for the Pref-1 mRNA in the precipitates by RT-PCR. As shown in the immunoblotting in Figure 4a, RNase-L protein was precipitated by specific monoclonal antibody against RNase-L (*α*-RNase-L), but not in the negative controls (Input and IgG_1_). The following RT-PCR experiments using RNA extracted from the immunoprecipitates showed specific Pref-1 amplicons from the precipitates with anti-RNase-L antibody, but not the negative controls, a specific amplicon of 36B4 (Figure 4b). Totally two separate amplicons of Pref-1 were used to detect its presence (Supplementary figure S1). The specificity of Pref-1 amplicons was confirmed by DNA sequencing (Supplementary Figures S2a and b). These data demonstrated the presence of Pref-1 mRNA in the protein complex containing RNase-L.

### Enzyme activity of RNase-L destabilized Pref-1 mRNA

If the Pref-1 mRNA is a substrate of RNase-L, the turnover rate of Pref-1 mRNA is supposed to be accelerated by the upregulation of RNase-L ribonuclease activity. Thus, we harvested the cells that were stably transfected with the plasmids with wild-type RNase-L, or its two mutants, C-terminal ribonuclease domain deletion mutant (ΔC151, deletion of amino acids 585–735)^4^ and Tyr711 to Ala single amino-acid substitution mutant (Y711A),^25^ and an empty vector at specific time points after actinomycin D treatment. Both the deletion and missense mutants of RNase-L gene were previously known to have reduced enzymatic activity.^25^ The RNase-L mRNA expression levels of the wild type and mutants were similar (Figure 4c). The Pref-1 turnover rate was significantly accelerated only in the stable clones overexpressed with the wild-type RNase-L (RNase-L), but not in those with mutants or empty vector (Figure 4d). As a control, GAPDH mRNA turnover rate was not affected by overexpression of either wild-type or mutant RNase-L (Figures 4e).

### Silencing of Pref-1 reversed the effect of RNase-L gene knockdown

**Figure 5:**
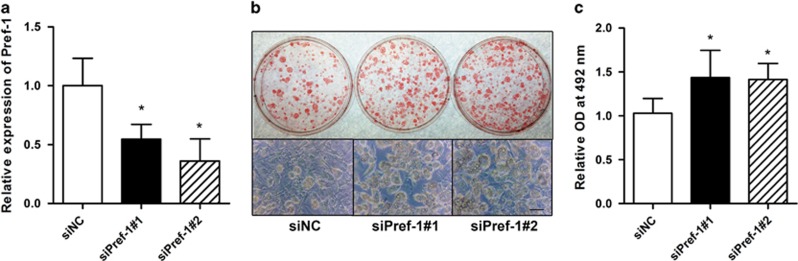
Silencing of Pref-1 increased adipocyte differentiation in RNase-L knockdown 3T3-L1 cells. (**a**) The silencing efficiencies of Pref-1 in siPref-1 #1 and #2 transfection compared to siNC transfection in RNase-L gene silencing stable clones of 3T3-L1 pre-adipocytes. Pref-1 expression was measured by qPCR. (**b**) After induction to differentiation, the lipid accumulation of the siPref-1 #1 and #2 transfected cells and siNC control cells were examined by Oil red O staining (upper) and bright field microscopy imaging (lower). Scale bar represents 50 μm. (**c**) The lipid content of 3T3-L1 in the experiments in (**b**) were quantified. The data represented in mean±S.E.M. were from three independent experiments. *P<0.05

To test the link between Pref-1 upregulation and adipocyte differentiation impairment upon RNase-L knockdown, we silenced the Pref-1 mRNA expression by siRNA in stable RNase-L knockdown 3T3-L1 pre-adipocytes and asked whether adipocyte differentiation could be rescued. The Pref-1 mRNA was silenced with siPref-1 #1 or #2, respectively, by 54.5 or 36.0%, in comparison to the off-target control, siNC (Figure 5a). After induction, the adipocyte differentiation was markedly increased (Figure 5b). The lipid content was significantly increased by approximately 40% irrespective of siPref-1 #1 or #2 compared to that of the controls (Figure 5c). This genetic epistasis analysis suggests that Pref-1 is down-stream to RNase-L and it is at least partially responsible for the effect of RNase-L downregulation on adipogenesis. However, additional factors cannot be excluded.

### The relationship between RNase-L and Pref-1 in mouse adipose tissues

**Figure 6:**
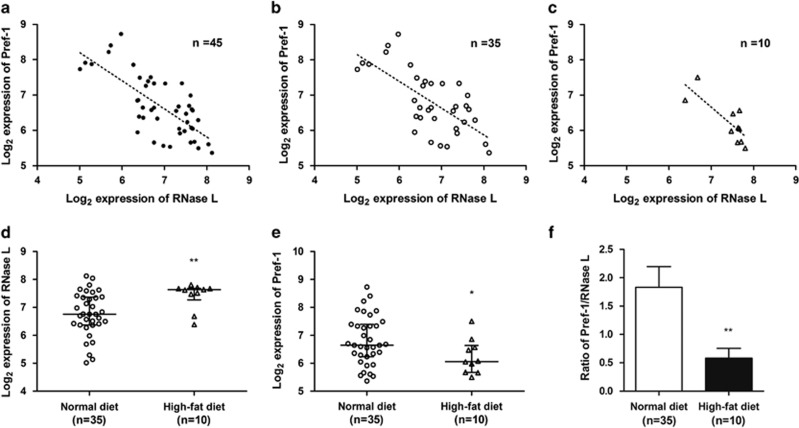
Negative correlation of mRNA expression between RNase-L and Pref-1 in normal mouse adipose tissues. Each solid dot represented the normalized RNase-L and Pref-1 expression at log_2_ scale of one mouse adipose tissue. The regression lines (dashed line) show the trend of Pref-1 change in gene expression with RNase-L within (**a**) whole arrays (*n*=45, *r*=−0.72, *P*=1.88 × 10^−8^), (**b**) ND (*n*=35, *r*=−0.69, *P*=5.59 × 10^−6^) and (**c**) HFD(*n*=10, *r*=−0.80, *P*=6.03 × 10^−3^) mouse data. The expression distributions comparison between ND and HFD of (**d**) RNase-L (HFD was 43.2% higher than ND, *P*=3.68 × 10^−3^), (**e**) Pref-1 (HFD was 38.8% lower than ND, *P*=0.030) and (**f**) the ratio of that Pref-1 divided by RNase-L (HFD was 68.1% lower than ND, *P*=3.78 × 10^−3^) is shown. Black circles=whole array; white circles=ND; white triangles=HFD. The expression distributions and ratio shown are in the scatter dot plot with median and interquartile range and in the bar chart with mean and S.E.M. *P<0.05 and **P<0.01

We have shown that RNase-L modulated the mRNA levels of Pref-1 in mouse 3T3-L1 cells. It seemed reasonable to expect a negative relationship between the expression level of RNase-L and that of Pref-1 in mouse adipose tissues. We answered this question by utilizing the public database. Among 45 array data from seven independent studies (Supplementary Table S1), there existed a significant negative relationship between RNase-L and Pref-1 mRNA levels in the mouse adipose tissues (*r*=−0.72, *P*=1.88 × 10^−8^, Figure 6a), even in the separate normal diet-fed (ND, *r*=−0.69, *P*=5.59 × 10^−6^, Figure 6b) or high-fat diet-fed (HFD, *r*=-0.80, *P*=6.03 × 10^−3^, Figure 6c) obese subgroup.

Interestingly, HFD obese subgroup seemed to have lower Pref-1 and higher RNase-L expressions (Figure 6c) in comparison to those of ND mice (Figure 6b). The RNase-L mRNA level in the adipose tissues of the HFD mice was 43.2% higher than that of ND mice (*P*=3.68 × 10^−3^, Figure 6d). In contrast, the mRNA level of Pref-1 was 38.8% lower in the adipose tissue of the HFD mice than that of ND mice (*P*=0.030, Figure 6e). As per the results, the Pref-1/RNase-L ratio was also 68.1% lower in the HFD subgroup than that in the ND mice (*P*=3.78 × 10^−3^, Figure 6f).

### Obese rats had higher RNase-L and lower Pref-1 expressions in adipose tissues

**Figure 7:**
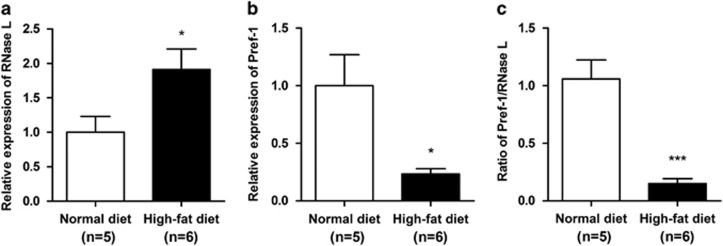
High-fat diet-induced obese rats (HFD) had higher RNase-L and lower Pref-1 expression in the adipose tissues compared with ND-fed rats. (**a**) The mRNA levels of RNase-L were 91.2% higher in HFD (*n*=6) rats than in ND (*n*=5), *P*=0.039. (**b**) The mRNA levels of Pref-1 were 76.5% lower in HFD than ND, *P*=0.046. (**c**) The Pref-1/RNase-L ratio was 85.0% lower in HFD than ND, *P*=4.36 × 10^−3^. The mRNA levels were determined by qPCR with TaqMan probe. The data shown are mean±S.E.M. *P<0.05 and ***P<0.001

In addition to computational analysis of mouse adipose tissues, we examined the adipose tissues from rats with different diets. The HFD-induced obese rats (*n*=6) had 1.9-fold higher RNase-L (*P*=0.039, Figure 7a) and only 23.5% Pref-1 (*P*=0.046, Figure 7b) mRNA levels in adipose tissues compared with the ND lean rats (*n*=5). The Pref-1/RNase-L ratio of the obese rats have only 15.0% that of the leans rats (*P*=4.36 × 10^−3^, Figure 7c).

## Discussion

**Figure 8:**
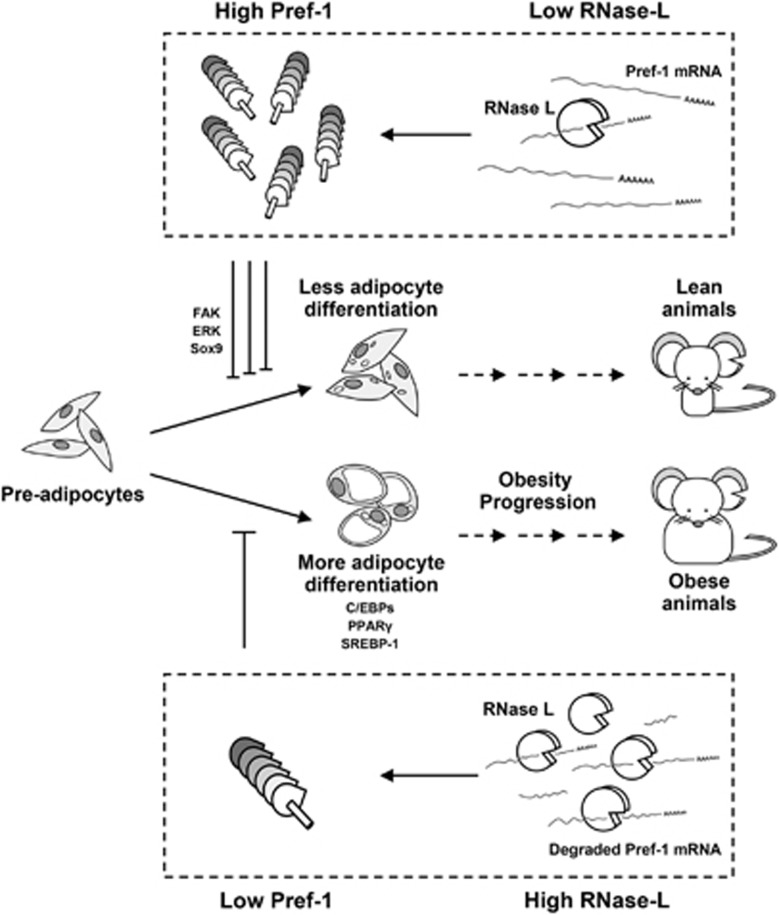
A proposed model of the role of RNase-L and Pref-1 in adipocyte differentiation and obesity progression. RNase-L protein interacts to Pref-1 mRNA and raises its turnover rate, followed by control of the downstream signals of Pref-1 that represses adipogenesis. This in turn regulates the differentiation of adipocytes and maturation of adipose tissues in animals

We herein reported that the adipocyte differentiation of 3T3-L1 was reduced by RNase-L knockdown and this inverse relation between RNase-L and Pref-1 in adipogenesis is also observed in obesity progression in animals (Figure 8). This effect is at least partially mediated by reduced breakdown of Pref-1 mRNA, a well-known negative regulator of adipogenesis in the very early phase of adipocyte differentiation.^22, 23^ This study probably is also the first report to implicate that Pref-1 mRNA is a substrate of RNase-L, and that, via modulating the expression of this new member of its substrate family, RNase-L participates in the regulation of adipocyte functions. This observation expands a recent view of RNase-L in addition to its classical roles in innate immunity.^4, 7, 26^

Our findings were consistent with the current concept of metainflammation,^14, 15^ suggesting that inflammation may not just be the results of obesity, but may also be a driver of adipogenesis. This vicious cycle may operate in the progression of obesity until it reaches a certain set point with a lower Pref-1/RNase-L ratio with higher inflammation and higher adipogenic potential. In fact, a recent report showed that injection of *Staphylococcus aureus* in subcutaneous adipose tissues of mouse promotes adipogenesis as a part of defense against bacterial invasion.^27^ Of further note, by selectively destabilizing the transcripts which are also regulated by certain micro-RNAs, RNase-L is capable of suppressing proliferation and adhesion in mammalian cells.^28^

We utilized 45 chip data from seven separate studies independent of our laboratory to investigate the relationship between RNase-L and Pref-1 expression in the mouse adipose tissues *in vivo*. Since RNase-L cannot be the only gene that regulates Pref-1 mRNA, a significant negative correlation with an *r* of −0.72 calculated using independent datasets submitted by different labs was indeed very impressive. On the other hand, a certain number of genes also had significant negative correlation with RNase-L mRNA. Whether these genes were directly regulated by RNase-L and whether they also participate in adipogenesis remain to be determined.

Previously, CHOP10 mRNA was also reported as an RNase-L substrate in primary immortalized mouse embryonic fibroblast (MEF) cells.^29^ CHOP10, also an inhibitory factor for adipocyte differentiation, was increased in RNase-L^−/−^ MEFs. However, in our study we did not observe significant changes in CHOP10 mRNA after RNase-L silencing in 3T3-L1 model. The discrepancy can possibly be explained by the following points: (1) our knockdown experiments did not abolish RNase-L expression completely like their knockout model; (2) the model system of MEFs is different from that of 3T3-L1.^29^ We also compiled the expression arrays of various tissues, in addition to adipose tissues, to perform meta-analyses. It is to be noted that CHOP10 mRNA indeed had significant negative correlation with RNase-L in MEFs (*n*=26, *r*=−0.67, *P*=2.03 × 10^−4^, Supplementary Figure S3d), but not in adipose tissues (*n*=45, *r*=−0.001, *P*=0.996, Supplementary Figure S3e) and embryos (*n*=13, r=0.71, *P*=7.08 × 10^−3^, Supplementary Figure S3c). In contrast to CHOP10, Pref-1 mRNA showed negative correlations with RNase-L during adipose development, including embryos (*n*=13, *r*=−0.79, *P*=1.48 × 10^−3^, Supplementary Figure S3a), MEFs (*n*=26, *r*=−0.76, *P*=6.37 × 10^−6^, Supplementary Figure S3b) and adipose tissues (Figure 6a and Supplementary Table S2).

One limitation of our study is that the mechanism was obtained in cells, but the *in silico* and *in vivo* data were a mixture of several cell types. We can only assume that the consistency between cell-line and tissue data was mainly derived from adipocytes. Whether other cell types in the adipose tissues also contribute to the negative relationship between RNase-L and Pref-1 remains to be investigated.

In summary, our data demonstrated that RNase-L is involved in adipogenesis through destabilizing Pref-1 mRNA, a novel substrate of RNase-L. By employing multiple approaches, including *in vitro* cell model, *in silico* array meta-analysis and *in vivo* animal data, we revealed the negative relationship between RNase-L and Pref-1 in mammals, and offer a new link among immunity, RNA metabolism, meta inflammation and adipogenesis.

## Materials and methods

### 3T3-L1 cell culture and differentiation

Mouse 3T3-L1 pre-adipocytes were purchased from the American Type Culture Collection and grown in Dulbecco's modified Eagle's medium (DMEM) with high glucose (4.5 g/l) supplemented with 10% (v/v) of newborn calf serum and 1% (v/v) of penicillin-streptomycin (Gibco, Waltham, MA, USA). For differentiation, confluent cells were cultured for additional 2 days and next cultured with induction medium of DMEM supplemented with 10% fetal bovine serum (FBS, Gibco), 1 *μ*M DEX (Sigma-Aldrich, St. Louis, MO, USA, D4902), 0.5 mM IBMX (Sigma-Aldrich, I7018) and 1 *μ*g/ml insulin. Forty-eight hours later, the induction medium was replaced with maintenance medium (DMEM with 10% FBS and 1 *μ*g/ml insulin) and the medium was changed every 2 days for 6–8 days until fully differentiated.

### RNase-L knockdown by lentiviral transduction

The RNase-L shRNA and control lentiviruses were obtained from Genomic Research Center, Academia Sinica (Taipei, Taiwan). For lentiviral transduciton, 3T3-L1 pre-adipocytes were seeded at approximately 40% confluence in 60 mm dishes (7 × 10^5^ cells) 1 day prior to transduction. On the day of transduction, 4 ml fresh medium containing polybrene (EMD Millipore, Billerica, MA, USA, TR-1003-G, final concentration [f.c.] of 10 ng/*μ*l) and RNase-L shRNA or control lentivirus at appropriate titers (8 × 10^6^ RIU virus, MOI=8) were added to each well. Forty-eight hours later, the infected cells were switched to medium containing puromycin (Invitrogen, Waltham, MA, USA, A11138-03, f.c.: 2 *μ*g/*μ*l) for selection for another 2 weeks. The stably transduced cells were induced to differentiation as described above.

### Oil red O staining

Oil red O (Sigma-Aldrich, O0625) stock solution (3.5 mg/ml in isopropanol) was prepared, mixed and stood at room temperature for 20 min followed by filtering with a 0.22 *μ*m filter. On the day of staining, the Oil red O working solution was freshly prepared (60% and 40% v/v stock and ddH_2_O).^30^ The differentiated 3T3-L1 cells were washed with PBS twice, fixed with 10% formaldehyde at room temperature for 10 min. After removal of the old formaldehyde, the cells were again fixed with 10% fresh formaldehyde for1 h, followed by washing twice with PBS and once with 60% isopropanol. After washing, the culture dishes were placed into a 50 °C oven for 15 min to dry the residual solvent completely. The cells were then stained with Oil red O working solution for 20 min at room temperature. To terminate the staining, the cells were washed four times with distilled water immediately following the removal of staining solution.

Lipid accumulation can be directly observed by bright field microscopy, and quantified by re-dissolving the Oil red O dye with 1 ml 100% isopropanol in each well; the OD values at 492 nm were measured by a spectrophotometer.

### RT-PCR and real-time PCR

Cells were washed with ice-cold PBS. Animal adipose tissues were homogenized by a homogenization gun. Total RNA was extracted using TRIzol reagent (Ambion, Waltham, MA, USA). For the synthesis of cDNA, reverse transcription (RT) was performed using a RT kit (Qiagen, Hilden, Germany) containing a genomic DNA (gDNA) wiping step, which can avoid the gDNA contamination in following PCR experiments. The resultant cDNA of 100 ng was amplified by semi-quantitative PCR (RT-PCR) with PCR master mix (Arrowtech, Rolla, MO, USA). PCR products were visualized in 1% agarose gel with ethidium bromide. In real-time PCR (qPCR) experiments, the cDNA of 25 ng was amplified by qPCR with Fast SYBR Green master mix and ABI StepOne Plus detection system (Applied Biosystems, Waltham, MA, USA), to quantify the relative mRNA levels for specific genes. For tissue samples, the Universal ProbeLibrary system (Roche, Penzberg, Germany) was used for cDNA synthesis. The cDNA was amplified and detected by FastStart TaqMan Probe Master (Roche) and LightCycler 480 instrument (Roche) with primer pair and TaqMan probe (Roche). The primer and probe sequences, and melting points (*T*_m_) of relevant genes for PCR are shown in Supplementary Table S3. Genes 36B4 was used as the reference gene in all experiments (no additional note), except that 18S rRNA was used in the assay of RNase-L expression trend during adipocyte differentiation (Figure 1a).

### Western blot analysis

Cells were washed with ice-cold PBS and lysed in lysis buffer (20 mM Tris-HCl, 150 mM NaCl, 1% Triton X-100, 1% sodium deoxycholate, 0.1% SDS in ddH_2_O) with protease inhibitor cocktail (Roche) and phosphatase inhibitor cocktail (Roche) to extract total cellular protein lysate. Protein concentration was determined by the Bradford protein assay using BSA as standard (Thermo Fisher, Waltham, MA, USA). For immunoblotting, cell lysate was boiled with 1 × sample buffer and 0.1 M dithiothreitol (Sigma-Aldrich) to denature protein. Boiled lysate of 10–50 μg was electrophoresed on a 10% SDS-PAGE gel in Tris-Glycine buffer system or a pre-cast 4–12% Bis-Tris gel (Thermo Fisher, NP0335BOX) in Novex NuPAGE MOPS system (Thermo Fisher, NP0001). The gel was next transferred onto a nitrocellulose membrane in Tris-Glycine-Methanol buffer. The membranes were blocked with 1 × Tris-buffered saline with Tween 20 (TBST) containing 5% BSA or nonfat milk, followed by incubation,respectively, withmouse anti-RNase-L monoclonal antibody (Abcam, Cambridge, UK, clone 2E9, ab13825, 1:1000), rabbit anti-Sox9 polyclonal antibody (Chemicon, Billerica, MA, USA, AB5535, 1:2000), rabbit anti-Pref-1 polyclonal antibody (Cell Signaling Technology, Danvers, MA, USA, #2069, 1:1000), rabbit anti-phospho-FAK monoclonal antibody (Cell Signaling Technology, Tyr397, clone D20B1, #8556, 1:1000), rabbit anti-FAK monoclonal antibody (Cell Signaling Technology, clone D2R2E, #13009, 1:1000), rabbit anti-phospho-ERK1/2 monoclonal antibody (Cell Signaling Technology, clone 197G2, #4377, 1:1000), rabbit anti-ERK1/2 monoclonal antibody (Cell Signaling Technology, clone 137F5, #4695, 1:1000) and rabbit anti-*β*-actin polyclonal antibody (GeneTex, Irvine, CA, USA, GTX109639, 1:10 000) as indicated in each experiment. Then the membranes were washed with 1 × TBST and incubated with horseradish peroxidase (HRP)-linked goat anti-mouse IgG polyclonal antibody (GeneTex, GTX213111-01, 1:20 000) or HRP conjugated donkey anti-rabbit IgG polyclonal antibody (Biolegend, San Diego, CA, USA, #406401, 1:10 000) as needed and washed thrice with 1 × TBST and the membranes were soaked in 1 × TBST until assays. The signals were quantified by an enhanced chemiluminescence detection system (GE Healthcare, Little Chalfont, UK).

### mRNP immunoprecipitation

The experimental procedure of mRNP immunoprecipitation (IP) was based on the protocol of Sanchez *et al.*^31^ Six to ten 150-mm dishes of 3T3-L1 pre-adipocytes were harvested and lysed with mRNP-IP (RIP) buffer (20 mM Tris-HCl, 50 mM NaCl, 2 mM EDTA, 1% NP40 in ddH_2_O) containing protease inhibitor cocktail and RNase inhibitor (Ambion, AM2694, 250 U/ml), then centrifuged at 10 000 ×  *g* to remove cell debris. Two mg of the lysate was incubated with 50 μl protein G magnetic beads (GE Healthcare, #10223458) in 1 ml RIP buffer at 4 °C for 2 h to purge non-specific binding to beads; the beads were then removed. For immunoprecipitating the ribonucleoprotein, the anti-RNase-L monoclonal antibody (Abcam, clone 2E9, ab13825, f.c.: 20 μg/ml), mouse IgG_1_ isotype control monoclonal antibody (BioLegend, clone MOPC-21, #400104, f.c.: 20 μg/ml) or without antibody was added to the cell lysate and gently agitated at 4 °C overnight with rotator. On the next day, 100 *μ*l beads were added and incubated at 4 °C for 4 h. The beads were recovered by magnetic block and washed thrice with ice-cold RIP buffer. The samples were made to stand on ice all the time. Each sample was separated into two equal portions, one for determining RNase-L protein using anti-RNase-L monoclonal antibody (Abcam, clone 2E9, ab13825, 1:1000) by western blotting and the other for RNA extraction with TRIzol reagent (Ambion). In the step involving RNA precipitation with isopropanol, glycogen (Ambion, AM9510, f.c.: 20 μg/ml), linear acrylamide (Ambion, AM9520, f.c.: 100 μg/ml) and sodium acetate (Sigma-Aldrich, W302406, f.c.: 0.3 M) were additionally applied to increase RNA recovery. The following experiments of western blotting, RT and RT-PCR were performed as described above.

### Pref-1 mRNA stability assay

The RNase-L-pcDNA3.1(-) plasmid was constructed by inserting the mouse RNase-L full-length (2.2 kb) cDNA, which was amplified from mouse transcriptome by PCR with specific primer pair (forward: 5′-CAAGGAAAAGGCATTGAGGA-3′ and reverse: 5′-AAAATGGTCCCATGAGCTTG-3′) into CMV-driven neomycin-resistant pcDNA3.1(-) vector (Invitrogen) vector using *Not*I (New England Biolabs, Ipswich, UK) restriction enzyme site. The ribonuclease domain of mouse RNase-L was inferred to locate on the amino acids 585–735 (homologous to human RNase-L amino acids 587–741),^4^ and the single amino-acid substitution of that Tyr711 (equivalent to human RNase-L Tyr712) was replaced with Ala, resulting in a significant decrease in enzyme activity.^25^ To generate a missense mutant, Y711A, the wild-type RNase-L-pcDNA3.1(−) was used as template for PCR with three specific primers: forward: 5′-ACCCAAGCTGGCTAGCATGGAGACCCCGGATTATAA-3′, reverse: 5′-GCCGCTCGAGTCTAGATCAGCTCTGTATGCCCCCTGGTCCCACTGCCTCTGGAACGCTGA-3′ and mutagenesis primer: 5′-actgcctctggaacgctgaggcgtggtggaggctgtgggaagtgttttctgGCttctgtt-3′. The targets of site-directed mutagenesis were T2131G and A2132C. To construct a C-terminal ribonuclease domain deletion mutant, *Δ*C151, the wild-type RNase-L-pcDNA3.1(-) was used as template for PCR with two specific primers: forward: 5′-ACCCAAGCTGGCTAGCATGGAGACCCCGGATTATAA-3′ and reverse: 5′-GCCGCTCGAGTCTAGATCAAAAGAAAGGATGGCCAA-3′. The two resultant mutant RNase-L cDNA were further cloned into pcDNA3.1(-) vector using Not I restriction enzyme site. The sequences of RNase-L mutant clones were confirmed by DNA sequencing.

The resultant plasmids or empty vector of 2 μg was individually transfected into 3T3-L1 pre-adipocytes at approximately 60% confluence in a 12-well plate (10^5^ cells per well) with 4 μl TurboFect transfection reagent (Thermo Fisher) in growth medium. Forty-eight hours post transfection, selection was performed with G418 at a concentration of 400 μg/ml for 2 weeks. The stable clones of pre-adipocytes were grown to confluence in six-well plates and treated with actinomycin D (Sigma-Aldrich, A9415) at a concentration of 10 μg/ml. The cells were harvested at various time points 0, 1, 2, 6, and 24 h for RNA extraction. RT and qPCR of Pref-1 and GAPDH mRNA were performed as described above. The time-course of Pref-1 mRNA of wild-type and mutant RNase-L overexpressed and control cells were analyzed and compared.

### Pref-1 silencing using siRNA transfection

The validated mouse Pref-1 siRNA#1 (s64996, antisense region located at exon 6 of Pref-1), Pref-1 siRNA#2 (s64998, antisense region located at exon 3 of Pref-1) and a non-targeting siRNA as the negative control were obtained from Ambion Silencer Select Pre-designed siRNA library (Ambion). Two distinct exon targets of siPref-1 were applied for verifying that the effect was not due to the siRNA sequence specificity. Following the manufacturer's instructions, on the previous day, 3T3-L1 pre-adipocytes were seeded in six-well plates at approximately 60% confluence (6 × 10^5^ cells per well). The siRNAs at f.c. of 100 nM (in Opti-MEM) were pre-mixed with 5 μl lipofectamine 2000 (Invitrogen) in 500 μl Opti-MEM medium (Invitrogen,). Then the mixture was added into each well. Six hours post transfection, the medium containing siRNA was replaced with fresh medium. Two days post confluence, the transfected cells were induced to differentiate as described above.

### Meta-analysis of mouse adipose tissue array datasets

Gene expression arrays of normal mouse adipose tissues were collected from Gene Expression Omnibus. All of the available raw data are generated by Platform GPL1261 of Affymetrix (Santa Clara, CA, USA) Mouse Genome 430 2.0 Array. Datasets with any gene manipulations or chemical treatments were eliminated. Forty-five arrays containing treatments with placebo, ND and high-fat diet from different types or locations of adipose tissues were included (Supplementary Table S1). These 45 arrays were further separated into two sub-datasets, ND (*n*=35) and HFD(*n*=10).

The data analyses were performed using the statistical software R (http://www.r-project.org). To compare the measurements of gene expression among chips from different datasets, raw data were background-corrected, quantile-normalized, log-transformed and summarized to the probe intensities using the robust multi-array average algorithm,^32, 33, 34^ which was implemented in the R package affy to ensure a similar empirical distribution of each array. If the probe sets were derived from the same gene, the expression intensity of this gene was represented by the average intensity of these probe sets.

### Animals and adipose tissue samples

Eleven 8-week-old male Sprague-Dawley rats were maintained under controlled environment without pathogens and,respectively, fed with ND (*n*=5, standard chow (5001, LabDiet, St. Louis, MO, USA)) or high-fat diet (*n*=6, DIO diet with 60% energy from fat (58Y1, TestDiet, St. Louis, MO, USA)) for 4 weeks. The adipose tissue samples were harvested from the abdominal fat, immediately snap-frozen in liquid nitrogen and stored at −80 °C for subsequent analysis. The experimental procedure was approved by the Institutional Animal Care and Use Committee in National Taiwan University.

### Statistical analysis

All data were calculated using Excel (Microsoft Office 2013, Redmond, WA, USA) and charted by Prism 5 (GraphPad Software Inc., San Diego, CA, USA). The data were generally presented as mean±S.D., mean±S.E.M. or median with interquartile range, as indicated. Differences between two groups of 3T3-L1 experiments were analyzed using two-tailed *t* test, and those of meta-analyses and animal data were analyzed using the two-tailed *t* test of Welch's correction. The relationship between RNase L and Pref-1 expression in meta-analysis was analyzed using the Pearson's correlation. A *P*<0.05 was considered significant.

## Abbreviations

RNase-L: Ribonuclease L
IFN: Interferon
OAS: Oligoadenylate synthetase
RIG-I: Retinoic acid-inducible gene 1
2-5A: (ppp5'A[2'p5'A]n)
IL: Interleukin
TNF: Tumor necrosis factor
HuR: Hu Antigen R
TTP: Tristetraprolin
Pref-1: Pre-adipocyte factor-1
C/EBP: CCAAT-enhancer-binding protein
PPARγ: Peroxisome proliferator-activated receptor γ
SREBP-1: Sterol regulatory element-binding protein-1
ADQ: Adiponectin
KLF: Krüppel-like factor
FAK: Focal adhesion kinase
ERK1/2: Extracellular signal-regulated kinases1/2
Sox9: SRY-box 9
ND: Normal diet-fed
HFD: High-fat diet-fed
MEF: Mouse embryonic fibroblast
RT: Reverse transcription
gDNA: Genomic DNA
RT-PCR: Semi-quantitative PCR
qPCR: Real-time PCR
T_m_: Melting points
TBST: Tris-buffered saline with Tween 20
HRP: Horseradish peroxidase
IP: Immunoprecipitation
mRNP: Messenger ribonucleoprotein
RIP: mRNP-IP
GEO: Gene Expression Omnibus
RMA: Robust multi-array average
