# Supplementary material for: A link between adipogenesis and innate immunity: RNase-L promotes 3T3-L1 adipogenesis by destabilizing Pref-1 mRNA
Source: Cell Death Dis. 2016 Nov 10;7(11):e2458–. doi: 10.1038/cddis.2016.323 (PMC5260905; doi:10.1038/cddis.2016.323)
Supplement: Supplementary Table S1 [file cddis2016323x1.docx]

**Supplementary Table S1.**

List of GEO sample ID (GSM) and reference series number (GSE) compiled for the meta-analysis of adipose tissues. *High-fat diet mouse

| **Sample ID** | **Reference Series** | **Sample ID** | **Reference Series** |
| --- | --- | --- | --- |
| GSM198456 | GSE8044 | GSM1185105 | GSE48811 |
| GSM198457 |  | GSM1185106 |  |
| GSM198458 |  | GSM1185107 |  |
| GSM198496 |  | GSM1185108 |  |
| GSM198523 |  | GSM1185109 |  |
| GSM198545 |  | GSM1185110 * |  |
| GSM338983 | GSE13432 | GSM1185111 * |  |
| GSM338984 |  | GSM1185112 * |  |
| GSM338985 |  | GSM1185113 * |  |
| GSM338989 |  | GSM1185114 * |  |
| GSM338990 |  | GSM1237791 | GSE51080 |
| GSM338991 |  | GSM1237793 |  |
| GSM493477 | GSE19757 | GSM1237794 |  |
| GSM493478 |  | GSM1237795 |  |
| GSM493479 |  | GSM1237797 |  |
| GSM493480 |  | GSM1237798 |  |
| GSM707747 | GSE28598 | GSM1237801 |  |
| GSM707748 |  | GSM1237802 |  |
| GSM707749 * |  | GSM1237806 |  |
| GSM707750 * |  |  |  |
| GSM795608 * | GSE32095 |  |  |
| GSM795610 * |  |  |  |
| GSM795612 * |  |  |  |
| GSM795616 |  |  |  |
| GSM795617 |  |  |  |
| GSM795625 |  |  |  |
