# Supplementary material for: A link between adipogenesis and innate immunity: RNase-L promotes 3T3-L1 adipogenesis by destabilizing Pref-1 mRNA
Source: Cell Death Dis. 2016 Nov 10;7(11):e2458–. doi: 10.1038/cddis.2016.323 (PMC5260905; doi:10.1038/cddis.2016.323)
Supplement: Supplementary Table S2 [file cddis2016323x2.docx]

**Supplementary Table S2.**

List of GEO sample ID (GSM) and reference series number (GSE) compiled for the meta-analyses of MEFs and embryos. These data were collected from GEO as Materials and Methods described.

| **MEFs** | | **Embryos** | |
| --- | --- | --- | --- |
| **Sample ID** | **Reference Series** | **Sample ID** | **Reference Series** |
| GSM120234 | GSE5324 | GSM257052 | GSE10167 |
| GSM120244 |  | GSM257054 |  |
| GSM120254 |  | GSM257056 |  |
| GSM120263 |  | GSM475934 | GSE19194 |
| GSM120272 |  | GSM475935 |  |
| GSM155304 | GSE6789 | GSM475936 |  |
| GSM156795 |  | GSM475937 |  |
| GSM160088 | GSE6945 | GSM990998 | GSE40296 |
| GSM160104 |  | GSM990999 |  |
| GSM215651 | GSE8704 | GSM991000 |  |
| GSM276426 | GSE10902 | GSM1008501 | GSE41095 |
| GSM276427 |  | GSM1008502 |  |
| GSM276428 |  | GSM1008503 |  |
| GSM409451 | GSE16266 |  |  |
| GSM409454 |  |  |  |
| GSM451626 | GSE18064 |  |  |
| GSM451627 |  |  |  |
| GSM451628 |  |  |  |
| GSM451632 |  |  |  |
| GSM451633 |  |  |  |
| GSM451634 |  |  |  |
| GSM509574 | GSE20335 |  |  |
| GSM509575 |  |  |  |
| GSM621319 | GSE25257 |  |  |
| GSM621320 |  |  |  |
| GSM621321 |  |  |  |
