# Supplementary material for: A link between adipogenesis and innate immunity: RNase-L promotes 3T3-L1 adipogenesis by destabilizing Pref-1 mRNA
Source: Cell Death Dis. 2016 Nov 10;7(11):e2458–. doi: 10.1038/cddis.2016.323 (PMC5260905; doi:10.1038/cddis.2016.323)
Supplement: Supplementary Table S3 [file cddis2016323x3.docx]

**Supplementary Table S3.**

List of primer sequences and probes used for RT-PCR and real-time PCR. The Tm of mouse primers are all 60°C and that of rats are all 55°C.

| Target | Forward primer | Reverse primer | Probe |
| --- | --- | --- | --- |
| Mouse | | | |
| 18s rRNA | 5’-cttagagggacaagtggcg-3’ | 5’-acgctgagccagtcagtgta-3’ | No |
| 36B4 | 5’-agattcgggatatgctgttggc-3’ | 5’-tcgggtcctagaccagtgttc-3’ | No |
| RNase-L | 5’-gcgaacacatcaatgaggaa-3’ | 5’-gaggctgtgggaagtgtttt-3’ | No |
| RNase-L #2 (exon 4-5) | 5’-aggacctgcaaccacaaaac-3’ | 5’-gtagagaaccagccgtccaa-3’ | No |
| Pref-1 | 5’-gaaaggactgccagcacaag-3’ | 5’-cacagaagttgcctgagaagc-3’ | No |
| Pref-1 #2 | 5’-ctcttgctcctgctggcttt-3’ | 5’-aatccatactgggggtcaca-3’ | No |
| KLF6 | 5’-gagttcctccgtcatttcca-3’ | 5’-gtcgccattacccttgtcac-3’ | No |
| KLF2 | 5’-accaactgcggcaagacctaca-3’ | 5’-cgcatccttcccagttgcaa-3’ | No |
| KLF3 | 5’-tacaggagaaaagccgtacaaatg-3’ | 5’-tcatcagaccgagcgaacttc-3’ | No |
| KLF7 | 5’-cacttaaaggcccaccagagg-3’ | 5’-cactcgcatccttcccatg-3’ | No |
| C/EBPβ | 5’-ggggttgttgatgtttttgg-3’ | 5’-cgaaacggaaaaggttctca-3’ | No |
| C/EBPδ | 5’-ttccaaccccttccctgat-3’ | 5’-ctggagggtttgtgtttttcgt-3’ | No |
| CHOP10 | 5’-cggaacctgaggagagagtg-3’ | 5’-tcataccaggcttccagctc-3’ | No |
| KLF4 | 5’-acagccacccacacttgtgactat-3’ | 5’-gtaaggtttctcgcctgtgt-3’ | No |
| KLF5 | 5’-acccggatctggagaagcga-3’ | 5’-cccgtatgagtcctcaggtgagc-3’ | No |
| C/EBPα | 5’-gggtgagttcatggagaatgg-3’ | 5’-cagtttggcaagaatcagagca-3’ | No |
| PPARγ | 5’-aagagctgacccaatggttg -3’ | 5’-gctttatccccacagactcg -3’ | No |
| SREBP-1 | 5’-gatcaaagaggagccagtgc-3’ | 5’-tagatggtggctgctgagtg-3’ | No |
| KLF9 | 5’-gtttgcccctgtaagtagtaagtg-3’ | 5’-ggttcaggccattgtgtagac-3’ | No |
| KLF15 | 5’-cgagaagccctttgcctgca-3’ | 5’-atcgccggtgccttgacaac-3’ | No |
| GAPDH | 5’-acagtccatgccatcactgcc-3’ | 5’-gcctgcttcaccaccttcttg-3’ | No |
| ADQ | 5’-accaaaagggctcaggatgct-3’ | 5’-gtaaagcgaatgggtacatt-3’ | No |
| Rat | | | |
| 36B4 | 5'-tgatgcccagggaagacagg-3' | 5'-cacaatgaagcattttgggtag-3' | UPL#85 |
| RNase-L | 5'-gactaaggacctcgttcattgc-3' | 5'-cacatcccgaagcgttctat-3' | UPL#2 |
| Pref-1 | 5'-gccctgcgtgatcaatggtt-3' | 5'-cacagaagttgcccgagaag-3' | UPL#9 |
