# Supplementary material for: A link between adipogenesis and innate immunity: RNase-L promotes 3T3-L1 adipogenesis by destabilizing Pref-1 mRNA
Source: Cell Death Dis. 2016 Nov 10;7(11):e2458–. doi: 10.1038/cddis.2016.323 (PMC5260905; doi:10.1038/cddis.2016.323)
Supplement: Supplementary Figure Legends [file cddis2016323x4.docx]

**Supplementary figure legends**

**Supplementary figure S1**

Presence of another Pref-1 mRNA amplicon in RNase-L-mRNP complex. RT-PCR was performed using primer pair Pref-1 #2 to determine the another mRNA region of Pref-1 from the precipitates of α-RNase-L and IP negative controls (Input and IgG_1_). The specific 36B4 amplicon was used as the negative control. The PCR products were electrophoresed with 1% agarose gel and then these amplicons of Pref-1 were quantitated. **p*<0.05

**Supplementary figure S2**

The specificity confirmation of Pref-1 amplicons from α-RNase-L precipitate by DNA sequencing. (**a**) The sequencing data and alignment of the Pref-1 amplicon in figure 4b was shown. (**b**) The sequencing data and alignment of the Pref-1 amplicon in supplementary figure S1 was shown. Sequence alignments were analyzed using the Nucleotide BLAST tool of NCBI (National Center for Biotechnology Information) website (<https://blast.ncbi.nlm.nih.gov/>).

**Supplementary figure S3**

The correlations of mRNA expression between RNase-L and its potential substrates, Pref-1 and CHOP10, in mouse embryos, MEFs and adipose tissues. Each solid dot represented the normalized expression at log_2_ scale of one tissue sample. The regression lines (dashed line) showed the trends of Pref-1 change in gene expression with RNase-L within the arrays of (**a**) embryos (n=13, r=-0.79, *p*=1.48x10^-3^), (**b**) MEFs (n=26, r=-0.76, *p*=6.37x10^-6^); and the trends of CHOP10 change in the data of (**c**) embryos (n=13, r=0.71, *p*=7.08x10^-3^), (**d**) MEFs (n=26, r=-0.67, *p*=2.03x10^-4^) and (**e**) adipose tissues (n=45, r=-0.001, *p*=0.996).
