# Supplementary figures and images for: A link between adipogenesis and innate immunity: RNase-L promotes 3T3-L1 adipogenesis by destabilizing Pref-1 mRNA
Source: Cell Death Dis. 2016 Nov 10;7(11):e2458–. doi: 10.1038/cddis.2016.323 (PMC5260905; doi:10.1038/cddis.2016.323)

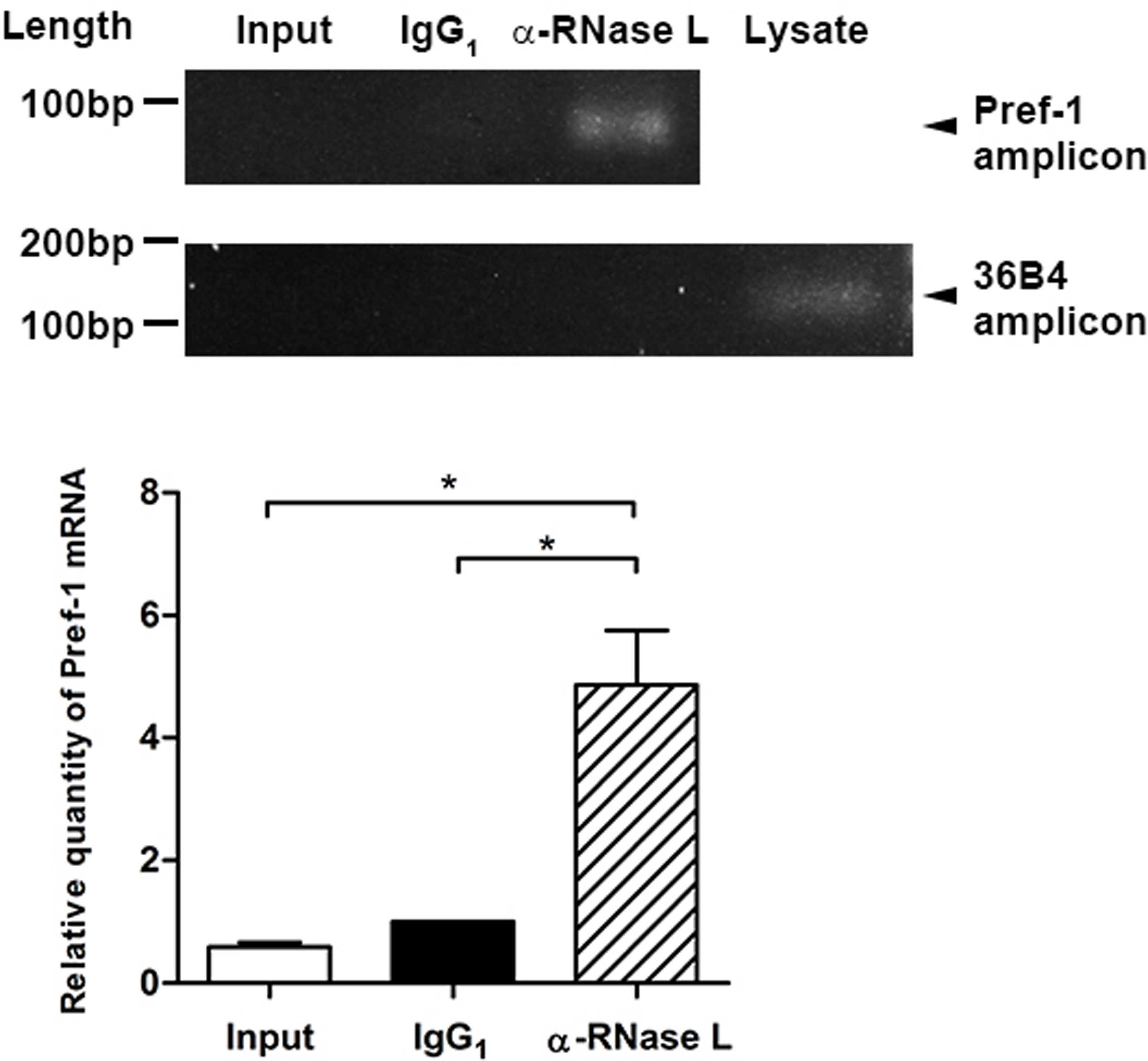

Supplement: Supplementary Figure S1 [file cddis2016323x5.tif]

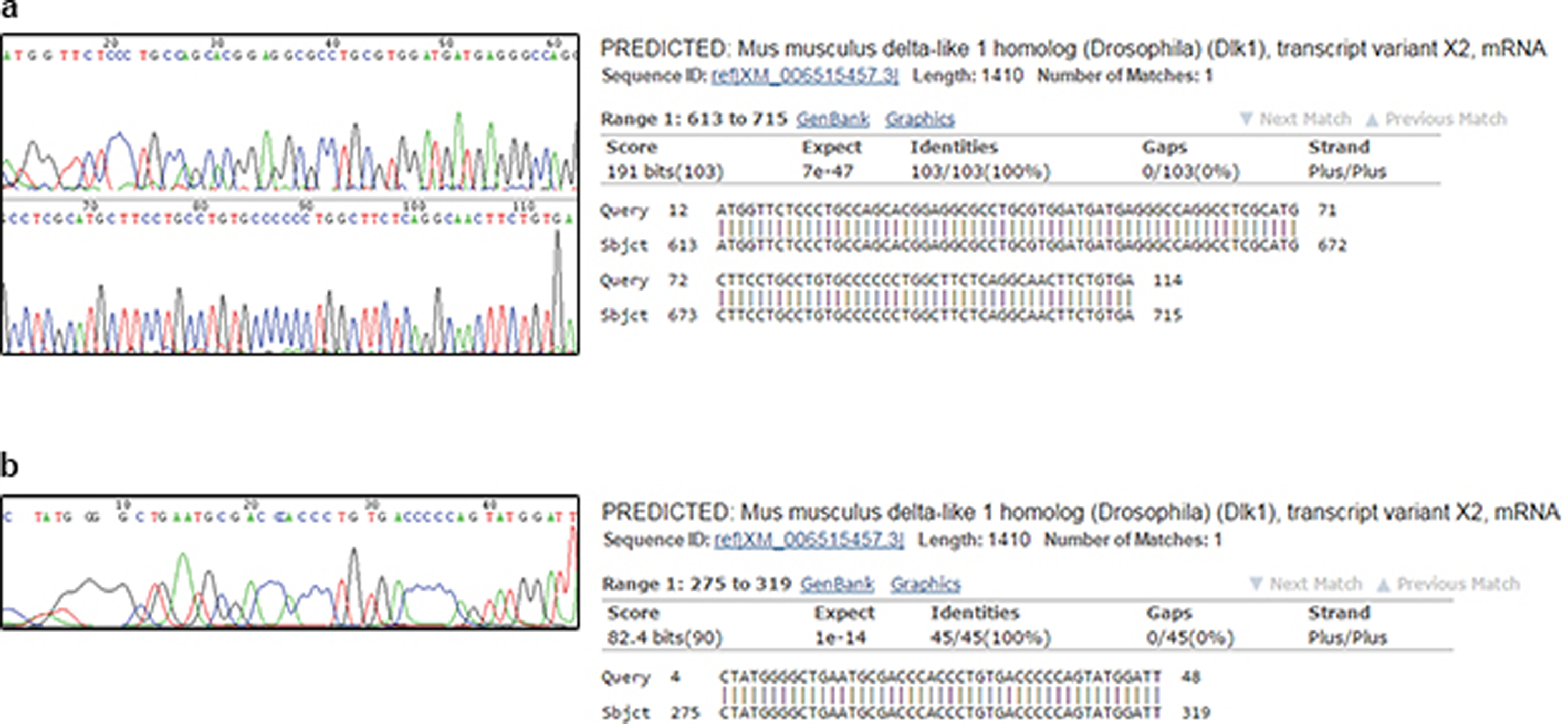

Supplement: Supplementary Figure S2 [file cddis2016323x6.tif]

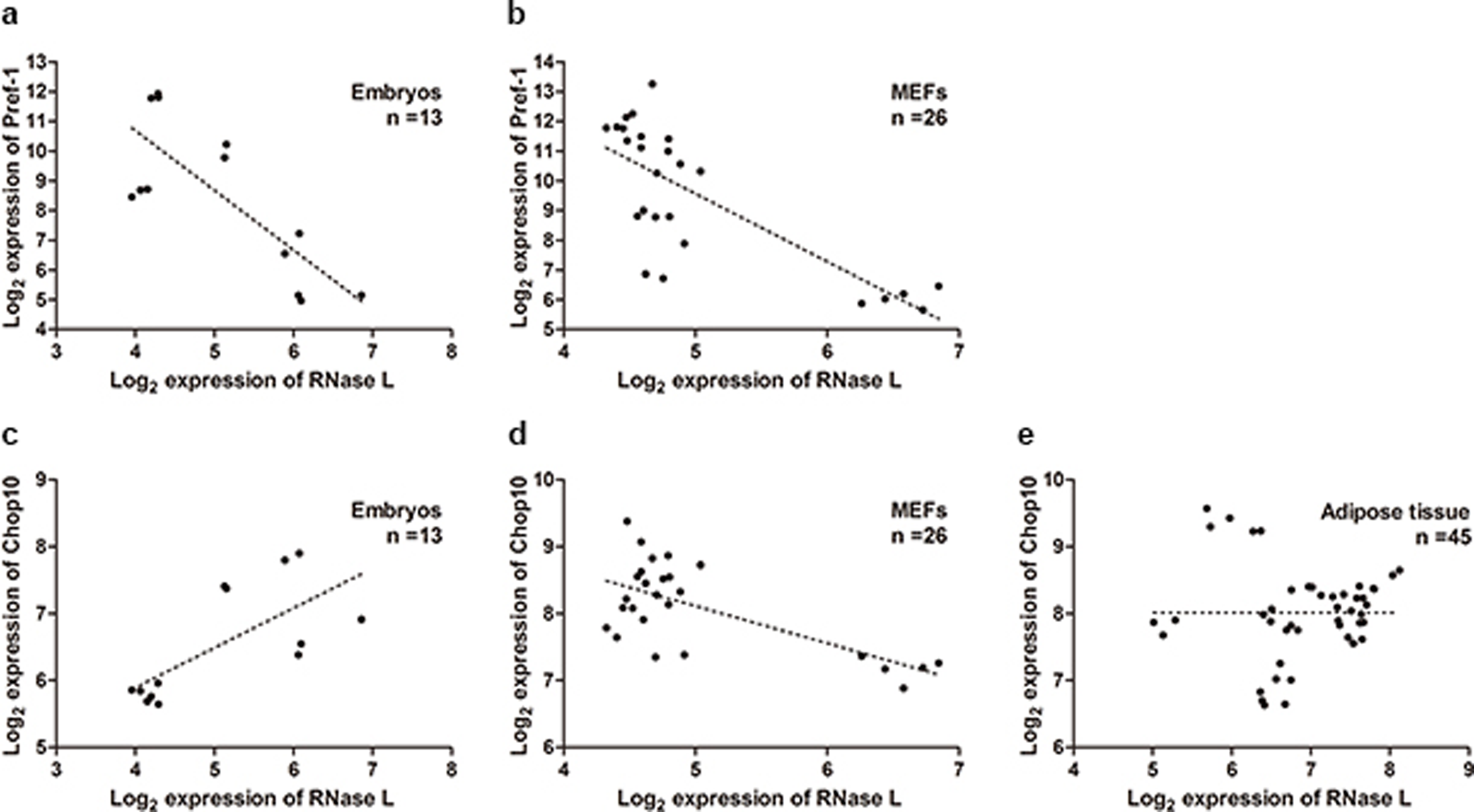

Supplement: Supplementary Figure S3 [file cddis2016323x7.tif]
